# Method for preparing DNA from feces in guanidine thiocyanate solution affects 16S rRNA-based profiling of human microbiota diversity

**DOI:** 10.1038/s41598-017-04511-0

**Published:** 2017-06-28

**Authors:** Koji Hosomi, Harumi Ohno, Haruka Murakami, Yayoi Natsume-Kitatani, Kumpei Tanisawa, Soichiro Hirata, Hidehiko Suzuki, Takahiro Nagatake, Tomomi Nishino, Kenji Mizuguchi, Motohiko Miyachi, Jun Kunisawa

**Affiliations:** 1 Laboratory of Vaccine Materials and Laboratory of Gut Environmental System, National Institutes of Biomedical Innovation, Health and Nutrition (NIBIOHN), Osaka, 567-0085 Japan; 2 Department of Physical Activity Research, National Institutes of Biomedical Innovation, Health and Nutrition (NIBIOHN), Tokyo, 162-8636 Japan; 3 Laboratory of Bioinformatics, National Institutes of Biomedical Innovation, Health and Nutrition (NIBIOHN), Osaka, 567-0085 Japan; 4 Department of Microbiology and Immunology, Kobe University Graduate School of Medicine, Hyogo, 650-0017 Japan; 5 Graduate School of Medicine, Graduate School of Pharmaceutical Sciences, Graduate School of Dentistry, Osaka University, Osaka, 565-0871 Japan; 6 Division of Mucosal Immunology, Department of Microbiology and Immunology and International Research and Development Center for Mucosal Vaccines, The Institute of Medical Science, The University of Tokyo, Tokyo, 108-8639 Japan

## Abstract

Metagenomic analysis based on the 16S rRNA gene is generally performed to examine the diversity and abundance of commensal bacteria in feces, which is now recognized to be associated with human health and diseases. Guanidine thiocyanate (GuSCN) solution is used as a less onerous way compared with a frozen method to transport and stock fecal samples at room temperature for DNA analysis; however, optimal methods to measure fecal bacterial composition in GuSCN solution remain to be investigated. Here, we examined the influence of various factors such as pretreatment (e.g., removing GuSCN solution and washing feces with phosphate-buffered saline (PBS) before mechanical lysis), fecal concentration in the GuSCN solution, storage time, and position of fecal subsampling on the 16S rRNA-based analysis of fecal bacteria in GuSCN solution. We found that pretreatment and fecal concentration affected the bacterial composition, and a little change was noted with subsampling position. Based on these results, we propose a basic protocol, including fecal sampling, sample storage, and DNA extraction, for the 16S rRNA-based analysis of bacterial composition in feces suspended in GuSCN solution.

## Introduction

Gut commensal bacteria are now recognized as an important factor in the maintenance of health because they facilitate food digestion and concomitant metabolite generation^1^, regulate the immune system^2^, and inhibit infection by potentially pathogenic microorganisms by competing for niches^3^. Technical advances in the deep sequencing of highly conserved regions in the 16S ribosomal RNA (rRNA) gene provide a convenient platform for the comprehensive and comparative analysis of the composition of commensal bacteria, including those that cannot currently be cultured^4, 5^. The results of such sequencing analyses reveal that altered composition of commensal bacteria frequently associates with the development and/or status of inflammatory immune diseases (e.g., Crohn’s disease and celiac disease) and allergies^6, 7^ as well as non-immune diseases such as obesity^8^, irritable bowel syndrome^9^, and diabetes^10^.

The bacterial composition in the gut has been evaluated mainly by using fecal samples, which are easy and non-invasive to obtain. Many factors during analysis affect the calculated composition of gut microbiota, including sample storage (frozen or room temperature; solution composition), homogenization method for DNA extraction (e.g., mechanical or enzymatic)^11, 12^, and choice of PCR primers^13^. For fecal storage, guanidine thiocyanate (GuSCN) is frequently used as a simple and safe means of storing feces at room temperature because GuSCN inactivates various nucleases^14–16^. The content of GuSCN solution is 100 mM Tris–HCl (pH 9), 40 mM EDTA, 4 M guanidine thiocyanate, and 0.001% bromothymol^17^. Generally, the use of GuSCN solution is combined with mechanical lysis using beads, followed by silica column purification. Although the DNA extraction method^18^ and design of PCR primers^19, 20^ has been extensively evaluated, optimization of the pre-treatment of fecal samples in GuSCN solution before mechanical lysis has not been well examined. In addition, procedures for the optimal storage of fecal samples in GuSCN solution remain to be examined. Here, we addressed these issues by comparing the data on the bacterial composition in fecal samples treated by various procedures, and validated whether the methods were truly optimized.

## Results and Discussion

### Effects of supernatant removal and washing of pellets on the microbial composition in GuSCN solution

**Figure 1:**
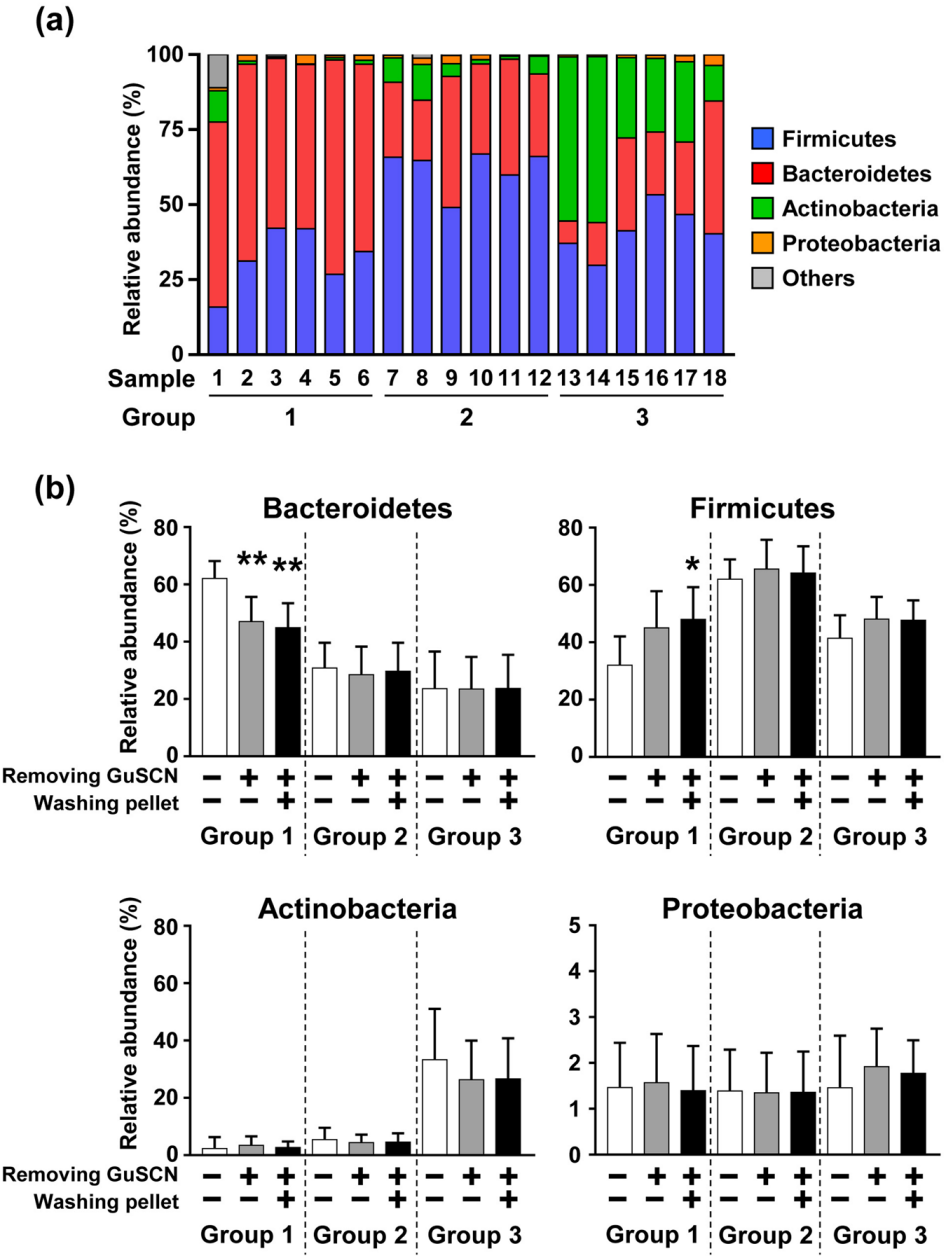
Effects of pretreatment on microbial composition in fecal samples stored in GuSCN solution. (**a**) Microbial composition of fecal samples from 18 healthy individuals at the phylum level. DNA was extracted directly from fecal samples in GuSCN solution by the bead beating method and sequenced. (**b**) Relative abundance of Bacteroidetes, Firmicutes, Actinobacteria, and Proteobacteria before and after pre-treatment in the DNA extraction procedure is shown. Data are presented as means ± SD (n = 6). An asterisk means statistically significant difference as calculated by using Student’s *t* test (*p < 0.05, **p < 0.01).

GuSCN solution is used as a simple way to transport and stock fecal samples at room temperature for DNA analysis; however, optimal methods for extraction of DNA from fecal samples in GuSCN solution to measure fecal bacterial composition remain to be investigated. In this study, we initially investigated whether pretreatment or no treatment (conventional method) before mechanical lysis affected microbial composition in feces suspended in GuSCN solution. To address this issue, we obtained fecal samples from 18 healthy individuals. Following a previous report^21^, the samples were divided into groups 1 to 3 based on the abundant bacteria at the phylum level: i.e., Actinobacteria, Firmicutes, and Bacteroidetes, respectively (Fig. 1a). Proteobacteria was a very minor component of the samples in all groups (Fig. 1a).

To address the effect of removal of GuSCN solution before mechanical lysis, GuSCN solution was removed by centrifugation and then DNA was extracted from the fecal pellets. Although this treatment did not affect the total number of operational taxonomic units (OTUs) when all groups were combined (Supplementary Fig. 1), the microbial composition appeared to be changed (Fig. 1b). Removing the GuSCN solution decreased the relative abundance of the predominant phylum Bacteroidetes and tended to increase the relative abundance of Firmicutes (p = 0.078) in group 1 (Fig. 1b and Supplementary Fig. 2). Group 2 (predominantly Firmicutes) did not show any marked changes in bacterial composition (Fig. 1b and Supplementary Fig. 2). Group 3 (abundantly Actinobacteria) did not show any marked changes as average (Fig. 1b), but some changes were observed in each individual; decrease of Actinobacteria in three individuals, decrease of Bacteroides in two individuals, and decrease in both Actinobacteria and Bacteroides in one individual were observed (Supplementary Fig. 2). Similar results were obtained when fecal pellets were further washed with PBS after removal of the GuSCN solution (Fig. 1b and Supplementary Fig. 2).

**Figure 2:**
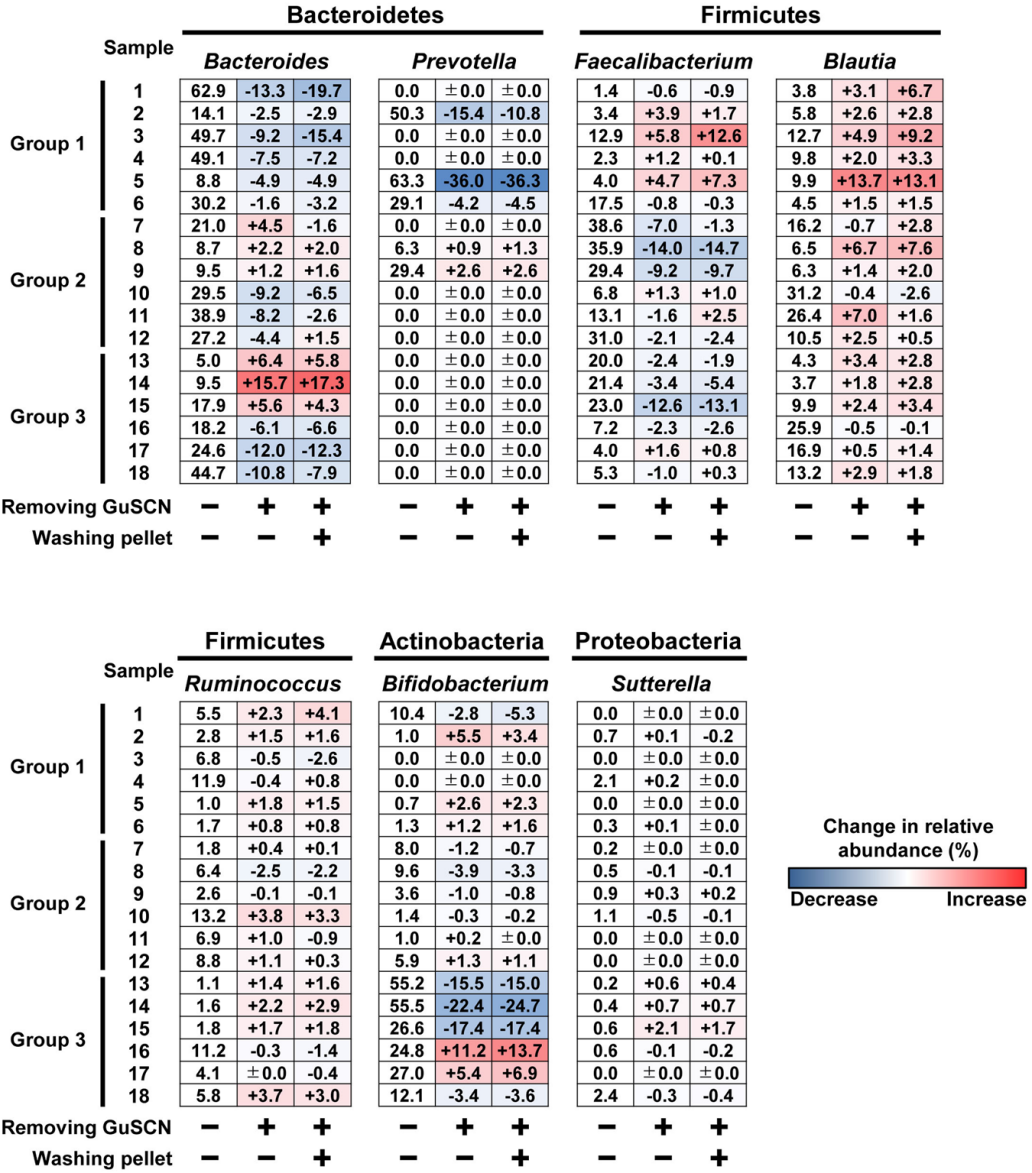
Effects of pretreatment on microbial composition in fecal samples at the genus level. The relative abundance (%) of *Bacteroides*, *Prevotella*, *Faecalibacterium*, *Blautia*, *Ruminococcus*, *Bifidobacterium*, and *Sutterella* in fecal samples collected from 18 individuals was shown. DNA was directly extracted from fecal samples in GuSCN solution (left column). The heat map (middle and right columns) shows the change in relative abundance (%) after pre-treatment in DNA extraction procedure.

We next performed a detailed analysis at the genus level. As mentioned above, in group 1, the dominant phylum Bacteroidetes decreased after pretreatment (Fig. 1b and Supplementary Fig. 2); at the level of genera, *Bacteroides* and *Prevotella* (in phylum Bacteroidetes) were reciprocally dominant in different individuals and tend to be decreased similarly after pretreatment with different significance among individuals (Fig. 2; Group 1). Although no marked differences at the phylum level between before and after pretreatment were noted in group 2 (Fig. 1b and Supplementary Fig. 2), there was a slight trend toward alteration of the bacterial composition of some genera in Firmicutes: with pretreatment there was a decrease in *Faecalibacterium* in some individuals, a marginal increase in *Blautia*, but no apparent change in *Ruminococcus* (*Faecalibacterium*, *p* = 0.421; *Blautia*, *p* = 0.661; *Ruminococcus*, *p* = 0.839) (Fig. 2; Group 2). *Bifidobacterium* was the predominant genus in the Actinobacteria phylum. Consistent with phylum data (Fig. 1 and Supplementary Fig. 2), some individuals with decreased ratio of Actinobacteria (#13–15) exhibited the decrease of *Bifidobacterium* (Fig. 2).

Because GuSCN is a chaotropic agent that denatures proteins (e.g., nucleases) and peptidoglycan, and lyses cells^22, 23^, each bacterium’s sensitivity to GuSCN may differ due to its cellular structure (e.g., cell morphology and size) and the components of its cell membrane and cell wall^24^. Indeed, in previous studies a different efficacy of DNA extraction was noted between gram-negative and -positive bacteria^12, 25, 26^. Thus, it is plausible that genomic DNA was released into the GuSCN solution from some bacteria (e.g., *Bacteroides* and *Bifidobacterium*) and discarded by pre-treatment procedures such as centrifugation and washing. To confirm this hypothesis, we examine whether genomic DNA of *Bacteroides* and *Bifidobacterium* was present in GuSCN solution by PCR using specific primers to each bacterial taxon. Genomic DNA of *Bacteroides* was detected in all samples of all groups (Supplementary Fig. 3a) and genomic DNA of *Bifidobacterium* was detected in some samples of groups 1 and 2, and in all samples of group 3 (Supplementary Fig. 3b). Therefore, we propose that DNA extraction should be performed directly from human fecal samples in GuSCN solution without pre-treatment. Expectedly, similar alteration may occur in shot-gun sequencing analysis of fecal samples in GuSCN solution.

### Changes in the bacterial composition in GuSCN solutions containing highly concentrated feces

**Figure 3:**
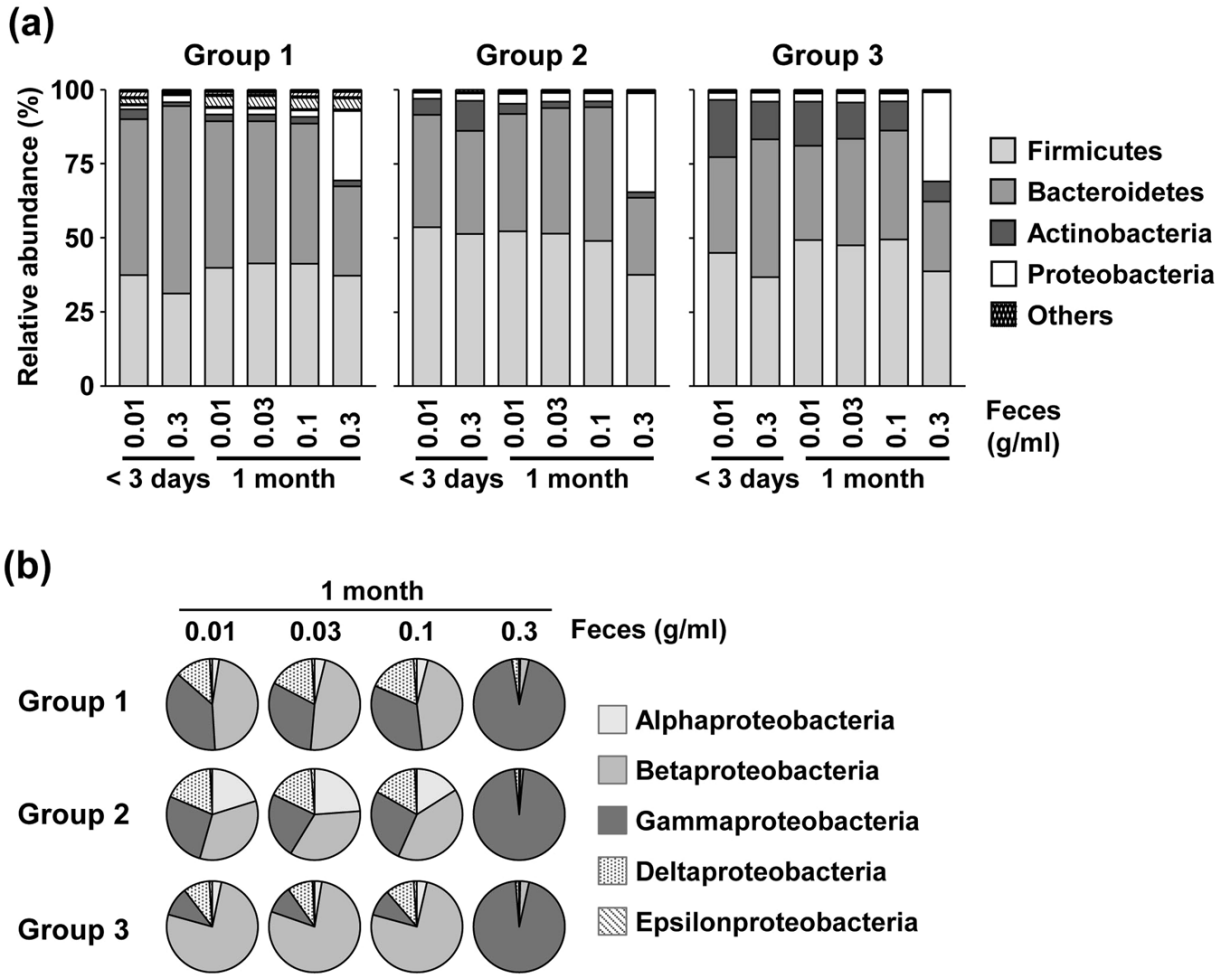
Effects of fecal concentration on microbial composition in fecal samples stored in GuSCN solution. (**a**) Microbial composition at the phylum level in GuSCN solutions containing different concentrations of feces (0.01–0.3 g/ml); Aliquots (0.01–0.3 g) of fecal samples were in 15 ml vials containing 1 ml of GuSCN solution. DNA was extracted within 3 days or after storage for one month at room temperature. (**b**) Class abundance of Proteobacteria after storage for one month at room temperature, graphed according to fecal concentration.

We next compared bacterial composition in GuSCN solutions with various concentrations of feces. At the phylum level, the fecal concentration did not affect the bacterial composition if the samples were analyzed within 3 days of storage (Fig. 3a and Supplementary Table 1). In contrast, when the samples were stored for 1 month, solutions with highly concentrated feces (0.3 g/ml) exhibited an increased relative abundance of Proteobacteria at the phylum level and Gammaproteobacteria at the class level in all three groups (Fig. 3a and b and Supplementary Table 1). When we performed a detailed analysis of Gammaproteobacteria at the genus level in each sample, we found that the proportions of several genera, such as *Citrobacter*, *Escherichia*, *Enterobacter*, and *Trabulsiella*, were moderately to highly increase in some samples, but not in others (Supplementary Fig. 4).

The main populations of commensal bacteria in feces are obligate anaerobes, which are primarily composed of the genera *Bacteroides*, *Eubacterium*, and *Clostridium* ^27^; obligate anaerobes cannot grow in aerobic conditions because they cannot use oxygen to generate ATP. Feces also contain facultative anaerobes as a minor population; these bacteria, which include members of Gammaproteobacteria, such as the orders Enterobacteriales, Vibrionales, and Pasteurellales^28^, can grow in both aerobic and anaerobic conditions due to their diverse capabilities of aerobic respiration, fermentation, and anaerobic respiration. Therefore, it is plausible that bacterial cells in highly concentrated feces (0.3 g/ml) are not inactivated sufficiently in GuSCN solution, leading to the observed increase in Gammaproteobacteria when samples were stored for one month under aerobic conditions. This hypothesis was also supported by PCR analysis using bacterium-specific primers; genomic DNA of *Citrobacter* was detected in highly concentrated samples stored for one month but not within 3 days (Supplementary Fig. 5).

**Figure 4:**
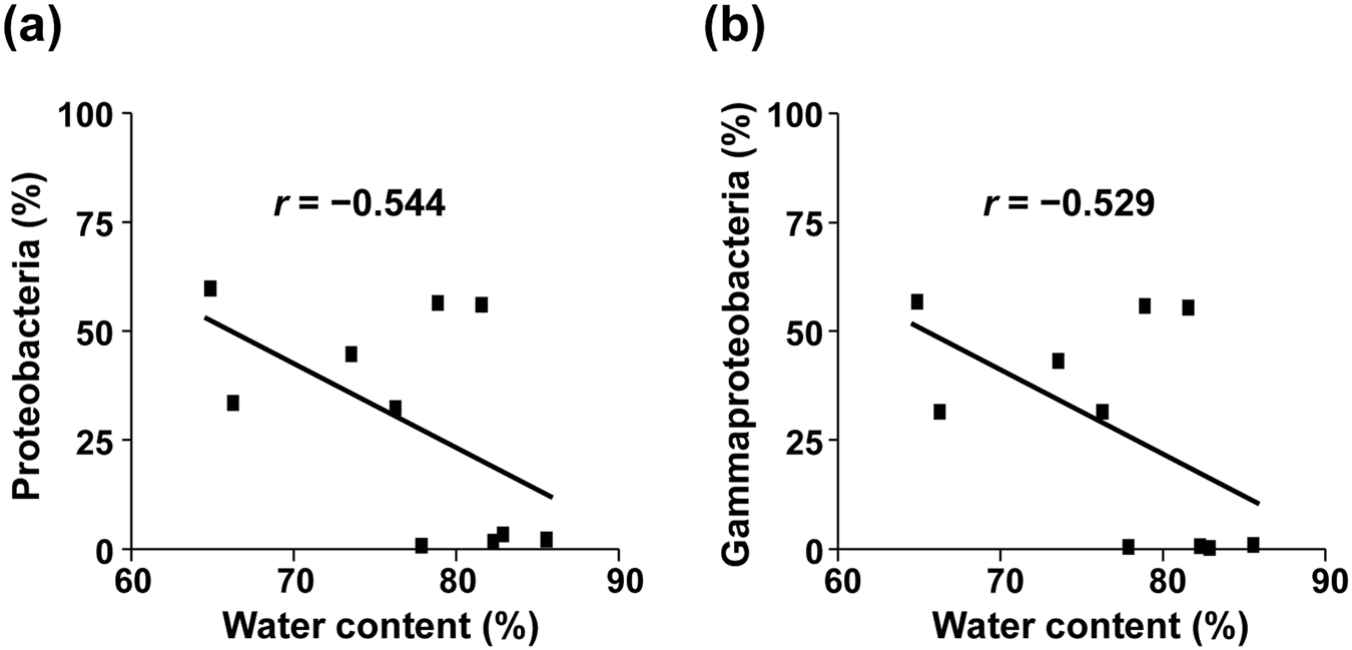
Relationship between water content in feces and the proportion of Proteobacteria and Gammaproteobacteria. The proportion of Proteobacteria (**a**) and Gammaproteobacteria (**b**) is shown for highly concentrated fecal samples (0.3 g/ml) in GuSCN solution after storage for one month at room temperature. *r*, Pearson correlation coefficient.

Additionally, we noticed that the increase in the relative proportion of Gammaproteobacteria in the samples with high fecal concentrations compared with low fecal concentrations occurred in all groups, but not in all individuals within each group (Supplementary Fig. 4). We therefore aimed to identify an individual-specific factor that determines the increase in Gammaproteobacteria. It was previously reported that the water content of a normal fecal sample is about 75%, while that of a diarrhoeic fecal sample is >85%, and that this difference affects the bacterial component^29^. In our experimental samples, the water content ranged between 64.9% and 85.6% (Supplementary Fig. 6). We found that the water content in feces was inversely correlated to the proportion of Proteobacteria at the phylum level, and the proportion of Gammaproteobacteria at the class level (Fig. 4a,b and Supplementary Fig. 6). These results suggest that highly concentrated feces (0.3 g/ml) with low water content were incompletely suspended in GuSCN solution, and therefore bacterial growth was not sufficiently inhibited.

### Dilution of GuSCN solution interferes with the maintenance of bacterial composition in feces

**Figure 5:**
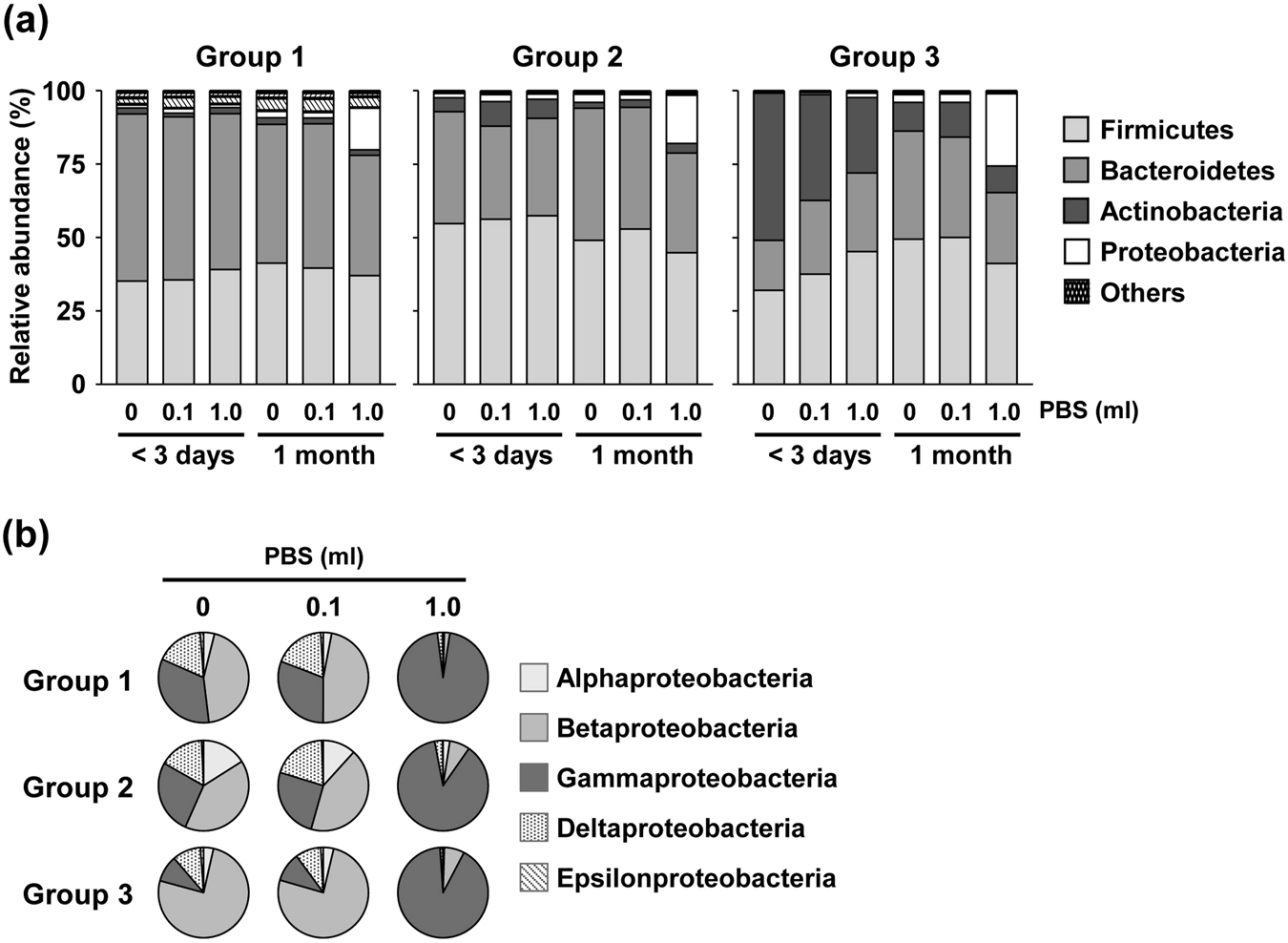
Effect of dilution of GuSCN solution on microbial composition in fecal samples. (**a**) Microbial composition at the phylum level for feces in GuSCN solution diluted with PBS; Aliquots (0.1 g) of fecal samples were in 15 ml vials containing 1 ml of GuSCN solution with 0, 0.1, or 1.0 ml of PBS. DNA was extracted within 3 days or after storage for one month at room temperature. (**b**) Abundance of various Proteobacteria at the class level in fecal samples after storage for one month at room temperature.

To explore this proposition of sample storage by using GuSCN solution further, we then compared fecal bacterial composition in GuSCN solutions diluted with various volumes of PBS. We used 0.1 g/ml as an experimental fecal concentration that has little effect on bacterial composition in the conventional method (Fig. 3a). The proportion of Proteobacteria increased at the phylum level when samples were diluted with 1.0 ml of PBS and stored for 1 month at room temperature (Fig. 5a and Supplementary Table 2). As was observed for GuSCN solutions with highly concentrated feces (0.3 g/ml) (Fig. 3), an increase in Proteobacteria was observed in all groups with sample dilution (Fig. 5a and Supplementary Table 2) and was coincident with the increase of the proportion of Gammaproteobacteria (Fig. 5b). Thus, we propose that insufficient bacteria killing by the diluted GuSCN solution led to the growth of Gammaproteobacteria in aerobic conditions.

We then performed a detailed analysis of each sample at the genus level and found that an increased proportion of several Gammaproteobacteria genera, such as *Citrobacter*, *Enterobacter*, and *Klebsiella*, in the samples diluted with PBS (Supplementary Fig. 7). Genomic DNA of *Citrobacter* was detected in the diluted samples stored for one month but not within 3 days by *Citrobacter*-specific PCR (Supplementary Fig. 8). As we discussed above, differences in bacterial structure may explain the differing effects on the inhibition of bacterial growth for different genera in the diluted GuSCN solution^24^.

In addition, the other conditions (e.g., dilution with 0.1 or 1.0 ml of PBS and storage for less than 3 days, and dilution with 0.1 ml of PBS for 1 month) affected the bacterial compositions in group 3, but not in group 1 or 2. Even when samples were stored for less than 3 days, dilution of samples decreased the relative proportion of Actinobacteria and simultaneously increased the relative proportions of Firmicutes and Bacteroidetes; similar but larger changes were observed with higher dilution (i.e., 1 ml of PBS) (Fig. 5a and Supplementary Table 2). A decrease in Actinobacteria and increase in Firmicutes and Bacteroidetes was also observed when non-diluted samples were stored for 1 month rather than less than 3 days (Fig. 5a and Supplementary Table 2). Therefore, we preformed further experiment to examine effect of storage term on bacterial composition of fecal samples in GuSCN solution. Consistently, the relative abundance of *Bifidobacterium* (predominant genus in Actinobacteria) appeared to decrease as time passes (Supplementary Fig. 9). These results suggest that the Actinobacteria DNA signal is easily lost when samples are stored for 2 to 4 weeks; it is possible that the release of DNA from Actinobacteria into GuSCN solution could lead to structural and/or biochemical changes that could affect PCR amplification and/or DNA extraction efficiency. Taken together, these results indicate that storage for one month at any temperature might lead to inaccurate bacterial composition data, especially for Actinobacteria-rich samples.

### Fecal sampling from several portions of same feces

**Figure 6:**
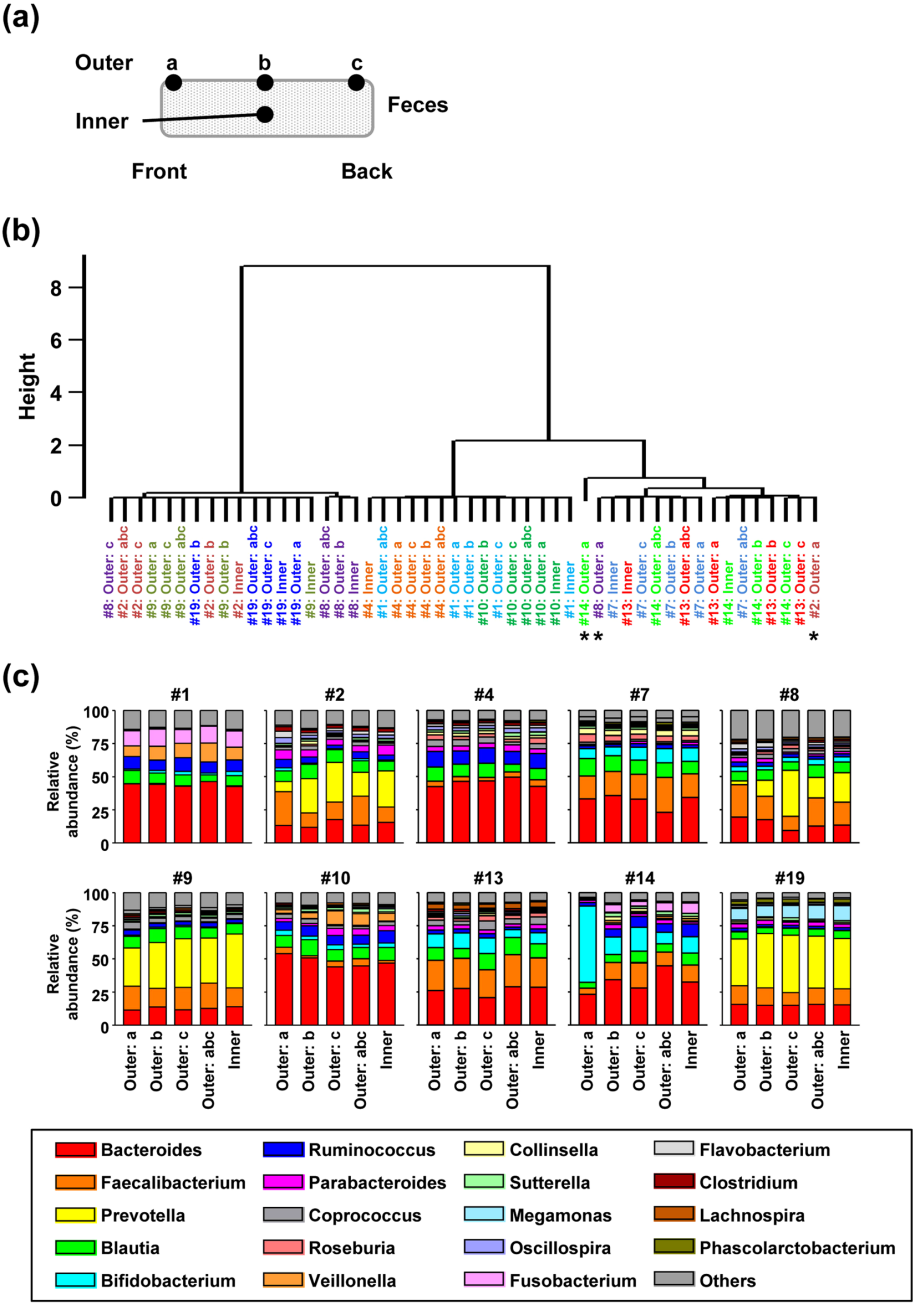
Comparison of the microbial composition at different positions in fecal samples. (**a**) Schematic diagram showing fecal sampling position. Subsamples were collected from different positions (outer or inner layer; front, middle, or back) in the feces. (**b**) Phylogenetic tree based on the microbial composition at the genus level. Samples collected from the same individual are indicated by the same color. The height represents relative genetic distance. The asterisks indicate samples that were clustered far from other samples collected from the same individual. (**c**) Microbial composition at genus level of different positions in feces from 10 individuals.

Finally, we examined whether bacterial composition was altered when we took subsamples from different positions (e.g., outer or inner; front, middle or back) within the feces (Fig. 6a). Overall, sampling position had little effect on the bacterial composition at the phylum level (Supplementary Fig. 10); however, phylogenetic analysis at the genus level indicated that subsamples from some individuals (e.g., #2, #8, and #14) showed differences between the outer layer at the front (indicated as “a” in Fig. 6a) and the other positions (Fig. 6b, indicated by asterisks). Additionally, a previous study by using more sensitive PCR analysis had shown that bacterial taxa abundance (e.g., Firmicutes and *Bifidobacteria* spp.) was different between the inside and outside of feces^30^. Because the bacterial composition obtained from a mixture of three positions (outer: “a”, “b”, “c” in Fig. 6a) appeared representative bacterial composition for each individual (Fig. 6b and c and Supplementary Fig. 10), fecal samples should be collected from several positions and mixed well before DNA extraction.

### Representative methods from fecal sampling to DNA extraction

**Figure 7:**
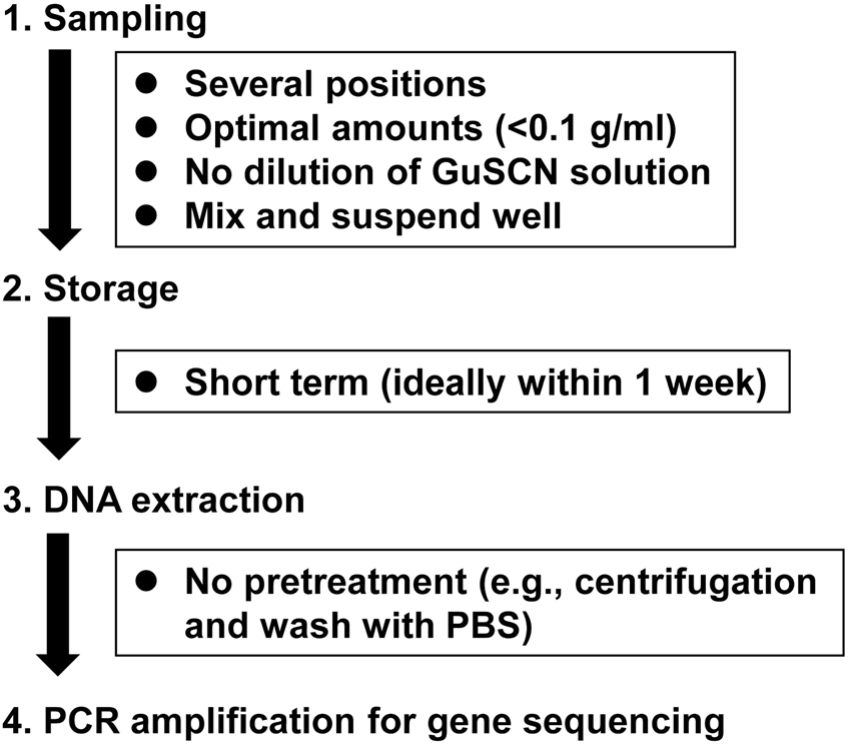
Schematic description of the proposed optimal protocol for 16S rRNA-based analysis of bacterial composition in fecal samples stored in GuSCN solution.

Our current findings suggest that the method using GuSCN solution is not a great solution for microbiome analysis if sampling and storage are not appropriately operated. Hence, the other methods such as liquid nitrogen homogenization technique can considered to be additional option. But, if researchers use GuSCN solution, we suggest the following protocol for representative extraction of DNA from bacterial populations in feces (Fig. 7):

1. Collect and pool subsamples from several positions on feces
2. To avoid growth of bacteria (especially Proteobacteria) during storage,

(i) make sure that the fecal concentration in the GuSCN solution is not too high;
(ii) suspend fecal samples, especially those with low water content, completely in the GuSCN solution

(3) Avoid long-term storage of feces to prevent reduction in the calculated proportion of Actinobacteria (ideally extract DNA within 1 week).
(4) Directly extract bacterial DNA from feces in GuSCN solution without any pretreatment.

## Materials and Methods

### Sample collection

Fecal samples were collected from totally 25 healthy adult volunteers (12 males: 22–55 years old; 13 females: 23–52 years old) at the National Institutes of Biomedical Innovation, Health and Nutrition (NIBIOHN), Osaka and Tokyo, Japan. All experiments were approved by the Committee of NIBIOHN and were conducted in accordance with their guidelines (approval numbers: kenei-14-1 and kenei-3) together with informed consent from all participants. Fecal samples from 10 individuals were used for the analyses in Figs 1–5, Supplementary Figs 1–9, and Supplementary Table 1, 2. Because two individuals withdrew from the study, an additional 2 individuals were recruited for the analyses in Fig. 6 and Supplementary Fig. 10. Furthermore, to generalize our results, additional 8 individuals were recruited for the analyses in Figs 1 and 2 and Supplementary Figs 1–3 and an additional 6 individuals were recruited for the analyses in Supplementary Fig. 9. Each fecal sample was mixed well and divided as follows: (1) some aliquots were placed in 15 ml vials containing 3 ml GuSCN solution (TechnoSuruga Laboratory Co., Ltd, Shizuoka, Japan)^17^, mixed by vortexing, and stored at room temperature (25 °C), (2) the other aliquots were placed in the vials containing GuSCN solution, phosphate-buffered saline (PBS) was added in some experiments, and the samples were mixed by vortexing, and stored at room temperature.

### DNA extraction

DNA was extracted from human fecal samples in GuSCN solution by the bead beating method. To remove GuSCN solution before mechanical lysis, 0.5 ml of fecal sample in GuSCN solution was centrifuged at 13,000 × *g* for 5 min at room temperature, the supernatant was removed, and the pellet was suspended in 0.5 ml of PBS. To wash the pellet, the suspension was centrifuged as above, the supernatant was removed, and the pellet was re-suspended in 0.5 ml of PBS. An aliquot (0.2 ml) of sample in GuSCN solution, the suspension, and the re-suspension were placed in 2-ml vials (WAKENBTECH Co., Ltd., Tokyo, Japan) containing 0.3 ml of lysis buffer (No. 10, Kurabo Industries Ltd., Osaka, Japan) and 0.5 g of 0.1-mm glass beads. The mixture was mechanically disrupted by bead beating using a Cell destroyer PS1000 (Bio Medical Science, Tokyo, Japan) at 4,260 rpm for 50 s at room temperature. The homogenized sample was centrifuged at 13,000 × *g* for 5 min at room temperature and 0.2 ml of the supernatant was collected, mixed with 0.15 ml of lysis buffer and 0.15 ml of proteinase K buffer (No. 2, Kurabo Industries Ltd) containing 0.4 mg/ml proteinase K, and DNA was extracted by using a Gene Prep Star PI-80X device (Kurabo Industries Ltd). The concentration of the extracted DNA was determined by using a NanoDrop Spectrophotometer ND-1000 (Thermo Fisher Scientific Inc., DE, USA), and samples were stored at −30 °C until use.

### 16S rRNA gene amplification and sequencing

The V3–V4 region of the 16S rRNA gene was amplified from the fecal DNA samples by using the following primers: forward: 5′-TCGTCGGCAGCGTCAGATGTGTATAAGCGACAGCCTACGGGNGGCWGCAG-3′, and reverse: 5′-GTCTCGTGGGCTCGGAGATGTGTATAAGAGACAGGACTACHVGGGTATCTAATCC-3′^20^. Reactions were carried out in 25-µl solutions containing 0.2 mM dNTPs, 1 mM MgSO_4_, 0.2 µM of each primer, 1 U KOD-Plus-v2 (Toyobo Co., Ltd., Osaka, Japan), 1 × PCR buffer for KOD-Plus, and 12.5 ng of sample DNA. The following thermal cycling conditions were used: initial denaturation at 95 °C for 3 min, followed by 25 cycles consisting of denaturation (95 °C for 30 s), annealing (55 °C for 30 s), and extension (68 °C for 1 min), and a final extension step at 68 °C for 5 min. PCR products were purified with 20 µl of Agecourt AMPure XP (Beckman Coulter, Inc., CA, USA) in accordance with the manufacturer’s protocol and eluted into 50 µl of 10 mM Tris-HCl, pH 8.5.

To prepare a DNA library for Illumina MiSeq using Nextera kit set A (Illumina Inc., CA, USA), PCR reactions were then performed in a 50 µl solution containing 0.2 mM dNTPs, 1 mM MgSO4, 5 µl of each primer, 1 U KOD-Plus v2, 1 × PCR buffer for KOD-plus, and 5 µl of the eluted DNA. The following thermal cycling conditions were used: initial denaturation at 95 °C for 3 min, followed by 8 cycles consisting of denaturation (95 °C for 30 s), annealing (55 °C for 30 s), and extension (68 °C for 1 min), and a final extension step at 68 °C for 5 min. PCR products were purified with 56 µl of Agecourt AMPure XP in accordance with the manufacturer’s protocol and eluted into 25 µl of 10 mM Tris-HCl, pH 8.5. The concentration of each PCR product was determined by using the QuantiFluor dsDNA System (Promega, Co., MI, USA); samples were diluted to a final concentration of 4 nM and pooled. The quality of the PCR products was analyzed by agarose gel electrophoresis. 16S rRNA gene sequencing of the PCR products was performed by using Illumina MiSeq (Illumina) in accordance with the manufacturer’s instructions.

### 16S rRNA sequence data analysis

Sequence reads from Illumina MiSeq were analyzed by using the Quantitative Insights Into Microbial Ecology (QIIME) software package^31, 32^. Twenty thousand paired-end reads (Phred quality score, >Q30) were selected from each sample. The selected reads were assembled with fastq-join and filtered by using split-libraries-fastq.py, and chimeric sequences were removed by using USEARCH v6.1. The sequences were clustered into OTUs on the basis of sequence similarity (>97%) using open reference OTU picking with UCLUST software. Taxonomy (phylum, class, order, family, genus, and species) and relative abundance were calculated by using the BaseSpace application 16S Metagenomics provided by Illumina and were based on the Greengenes database^33, 34^. Phylogenetic analysis was performed by using the R package with pvclust based on Pearson correlation coefficient and ward.D2 method (nboost = 10,000)^35^.

### Bacterium specific PCR

Bacterium specific PCR was performed by using primers specific for *Bacteroides* ^36^ (forward: 5′-AACGCTAGCTACAGGCTT-3′ and reverse: 5′-CAATCGGAGTTCTTCGTG-3′, annealing at 53 °C), *Bifidobacterium* ^37^ (forward: 5′-GGGTGGTAATGCCGGATG-3′ and reverse: 5′-CCACCGTTACACCGGGAA-3′, annealing at 62 °C), and *Citrobacter* ^38^ (forward: 5′-TTGGCGTCCAGCGCATTCA-3′ and reverse: 5′-AATTCCAGCCTTCGGCAAACG-3′, annealing at 60 °C). Reactions were carried out in 20-µl solutions containing 0.2 mM dNTPs, 1 mM MgSO4, 0.2 µM of each primer, 1 U KOD-Plus-v2 (Toyobo Co., Ltd.), 1 × PCR buffer for KOD-Plus, and 0.01 ng of sample DNA. The following thermal cycling conditions were used: initial denaturation at 94 °C for 2 min, followed by 35 (for *Bacteroides*) and 40 (for *Bifidobacterium* and *Citrobacter*) cycles consisting of denaturation (94 °C for 15 s), annealing (30 s), and extension (68 °C for 1 min), and a final extension step at 68 °C for 5 min. PCR product was detected by electrophoresis in a 1.5% agarose gel, stained with ethidium bromide, and visualized by transillumination by UV light. DNA molecular weight standard (Nacalai tesque, Inc., Kyoto, Japan) was analyzed along with the samples in each electrophoresis run.

## Electronic supplementary material

Supplementary Information
