# Supplementary material for: Method for preparing DNA from feces in guanidine thiocyanate solution affects 16S rRNA-based profiling of human microbiota diversity
Source: Sci Rep. 2017 Jun 28;7:4339. doi: 10.1038/s41598-017-04511-0 (PMC5489508; doi:10.1038/s41598-017-04511-0)
Supplement: Supplementary file 1 — Supplementary Information [file 41598_2017_4511_MOESM1_ESM.pdf]

Supplementary Information

Method for preparing DNA from feces in guanidine thiocyanate solution affects 16S rRNA-based profiling of human microbiota diversity

Koji Hosomi[1], Harumi Ohno[2], Haruka Murakami[2], Yayoi Natsume-Kitatani[3], Kumpei Tanisawa[2], Soichiro Hirata[1,4], Hidehiko Suzuki[1], Takahiro Nagatake[1], Tomomi Nishino[1], Kenji Mizuguchi[3], Motohiko Miyachi[2*], and Jun Kunisawa[1,4-6*]

1 Laboratory of Vaccine Materials and Laboratory of Gut Environmental System, National Institutes of Biomedical Innovation, Health and Nutrition (NIBIOHN), Osaka 567-0085, Japan

2 Department of Health Promotion and Exercise, National Institutes of Biomedical Innovation, Health and Nutrition (NIBIOHN), Tokyo 162-8636, Japan

3 Laboratory of Bioinformatics, National Institutes of Biomedical Innovation, Health and Nutrition (NIBIOHN), Osaka 567-0085, Japan

4 Department of Microbiology and Immunology, Kobe University Graduate School of Medicine, Hyogo 650-0017, Japan

5 Graduate School of Medicine, Graduate School of Pharmaceutical Sciences, Graduate School of Dentistry, Osaka University, Osaka 565-0871, Japan

6 Division of Mucosal Immunology, Department of Microbiology and Immunology and International Research and Development Center for Mucosal Vaccines, The Institute of Medical Science, The University of Tokyo, Tokyo 108-8639, Japan

*Correspondence to Jun Kunisawa (E-mail: kunisawa@nibiohn.go.jp) and Motohiko Miyachi (E-mail: miyachi@nih.go.jp)

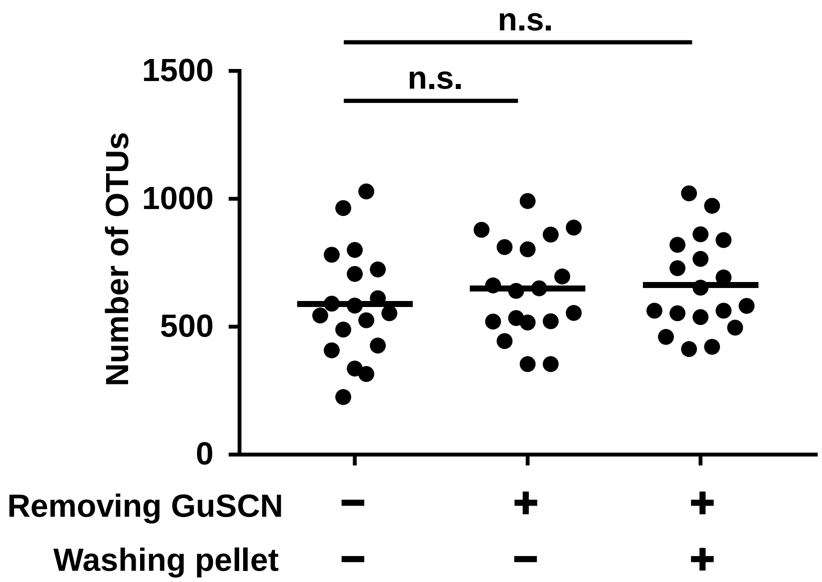

Supplementary Figure 1. Effects of pretreatment on the number of operational taxonomic units.

Operational taxonomic units (OTUs) were defined based on 97% similarity and a sequencing depth of 11,450 pyrotags. ‘n.s.’ means no statistically significant difference as calculated by using Student’s t test.

Group	Sample	Bacteroidetes			Firmicutes		
Group 1	1	61.7	-13.1	-19.6	15.8	+10.7	+19.0
	2	65.6	-16.9	-13.5	31.2	+10.8	+10.0
	3	56.6	-8.1	-13.3	42.1	+8.0	+13.5
	4	54.7	-5.8	-7.4	42.0	+5.8	+7.5
	5	71.4	-40.7	-41.0	26.8	+38.2	+38.8
	6	62.4	-5.7	-8.3	34.4	+4.6	+7.0
Group 2	7	25.0	+4.5	-2.8	65.8	-3.5	+3.6
	8	20.1	+0.8	+0.9	64.7	+5.0	+4.0
	9	43.7	+2.8	+2.8	49.0	-1.6	-1.8
	10	29.9	-9.7	-6.8	66.9	+10.6	+7.1
	11	38.6	-8.1	-2.5	59.9	+7.8	+2.5
	12	27.5	-4.3	+1.5	66.0	+2.9	-2.7
Group 3	13	7.4	+6.0	+6.5	37.1	+8.6	+7.5
	14	14.2	+17.0	+17.6	29.8	+4.2	+5.8
	15	30.9	+5.0	+3.5	41.3	+10.3	+12.1
	16	20.9	-6.8	-7.8	53.3	-4.2	-5.6
	17	24.2	-11.8	-12.1	46.7	+7.0	+6.0
	18	44.3	-10.4	-7.6	40.3	+14.5	+12.1

Group	Sample	Actinobacteria			Proteobacteria		
Group 1	1	10.4	-2.8	-5.3	1.0	+0.1	±0.0
	2	1.1	+5.5	+3.5	2.0	+0.6	+0.1
	3	0.2	±0.0	+0.1	0.4	±0.0	-0.1
	4	0.2	±0.0	±0.0	3.1	±0.0	-0.1
	5	0.8	+2.5	+2.3	0.7	±0.0	-0.1
	6	1.3	+1.2	+1.6	1.6	-0.1	-0.2
Group 2	7	8.1	-1.2	-0.7	0.9	+0.2	-0.1
	8	11.9	-5.4	-4.5	2.1	±0.0	-0.1
	9	4.3	-1.3	-1.0	2.7	±0.0	±0.0
	10	1.5	-0.3	-0.2	1.6	-0.7	-0.1
	11	1.0	+0.2	±0.0	0.5	+0.1	±0.0
	12	5.9	+1.3	+1.1	0.5	+0.1	+0.1
Group 3	13	54.7	-15.6	-14.9	0.7	+0.9	+0.9
	14	55.3	-22.3	-24.6	0.5	+0.9	+0.9
	15	26.7	-17.5	-17.5	1.0	+2.2	+1.8
	16	24.6	+11.1	+13.5	1.1	±0.0	-0.2
	17	26.8	+5.2	+6.7	2.0	-0.4	-0.6
	18	11.9	-3.3	-3.5	3.5	-0.8	-1.0
Removing GuSCN		–	+	+	–	+	+
Washing pellet		–	–	+	–	–	+

Change of relative abundance (%)

Decrease — Increase

Supplementary Figure 2. Effects of pretreatment on microbial composition in fecal samples in GuSCN solution.
The relative abundance (%) of Bacteroidetes, Firmicutes, Actinobacteria, and Proteobacteria in fecal samples collected from 18 individuals. DNA was directly extracted from fecal samples in GuSCN solution (left column). The heat map in middle and right columns shows the change in relative abundance (%) after pre-treatment in the DNA extraction procedure.

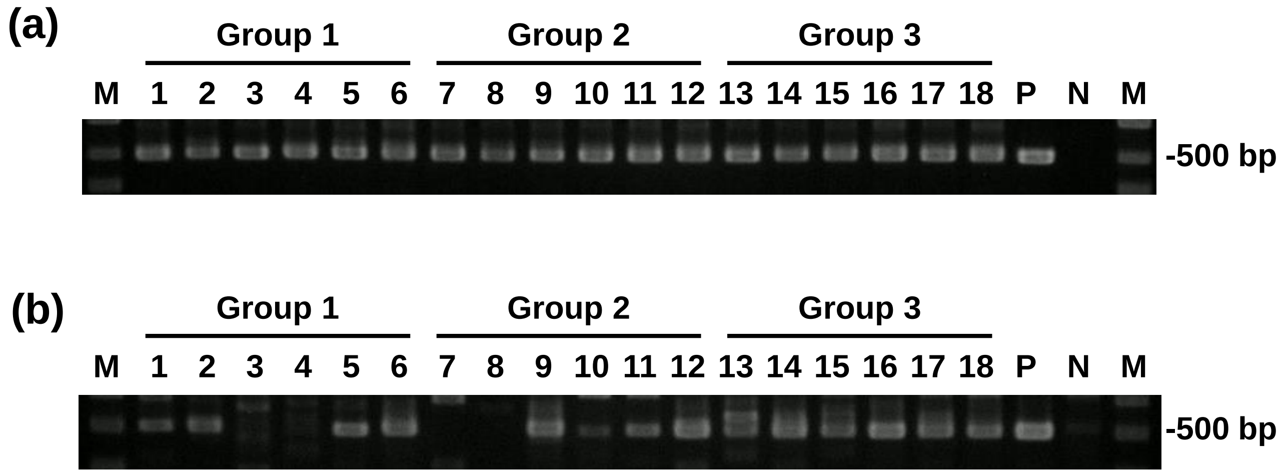

Supplementary Figure 3. Detection of genomic DNA of *Bacteroides* and *Bifodobacterium* in GuSCN solution by PCR.

DNA was extracted from the supernatants after centrifugation fecal samples in GuSCN solution by the bead beating method. PCR was performed using primers specific for *Bacteroides* (a) and *Bifidobacterium* (b). M, 1 kbp DNA ladder one. P, genomic DNA extracted from Bifidobacterium lungum or Bacteroides vulgatus as a positive control. N, genomic DNA extracted from *Escherichia coli* as a negative control.

	Sample	*Citrobacter*	*Esherichia*	*Enterobacter*	*Trabulsiella*	*Serratia*	*Klebsiella*	Others/ unclasified
Group 1	1	0.0	0.0	0.0	0.0	0.0	0.0	0.7
	2	0.0	22.3	0.6	0.0	3.8	0.0	3.9
	3	0.0	0.2	0.0	0.0	0.1	0.0	0.1
	4	3.5	0.2	9.2	23.8	1.4	0.1	17.9
Group 2	7	31.0	0.1	14.2	0.4	0.1	1.0	8.0
	8	16.2	0.0	10.8	0.6	0.1	1.7	12.1
	9	0.0	0.0	0.0	0.0	0.0	0.0	0.4
Group 3	13	0.0	11.3	4.5	0.1	5.9	0.1	8.4
	14	31.7	0.0	12.5	0.5	0.0	3.9	6.6
	15	0.0	0.0	0.0	0.0	0.0	0.0	0.1

Relative abundance (%): low — high

Supplementary Figure 4. Proportion of dominant genera of Gammaproteobacteria in each fecal sample.
The heat map shows the genus abundance (%) of Gammaproteobacteria in highly concentrated fecal samples (0.3 g/ml) in GuSCN solution after storage for one month at room temperature.

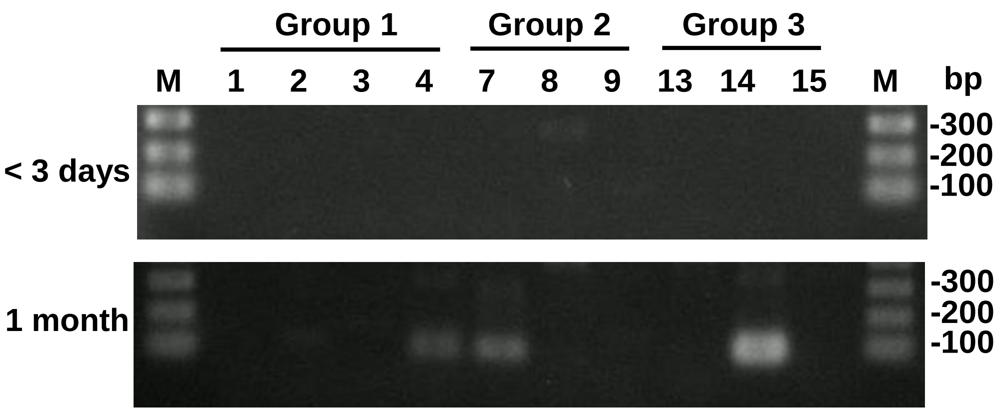

Supplementary Figure 5. Detection of genomic DNA of Citrobacter in highly concentrated fecal samples by PCR. Citrobacter-specific PCR was performed using DNA extracted from highly concentrated fecal samples (0.3 g/ml) in GuSCN solution within 3 days and after strage for 1 month at room temperature. M, 100 bp DNA ladder.

	Sample	Water Content (%)	Proteobacteria		Gammaproteobacteria	
			< 3 days	1 month	< 3 days	1 month
Group 1	1	85.6	0.2	2.0	0.1	0.8
	2	66.3	6.6	33.4	4.3	31.4
	3	77.9	0.6	0.6	0.4	0.4
	4	64.9	4.0	59.6	0.3	56.6
Group 2	7	81.6	0.7	55.9	0.3	55.3
	8	73.6	2.7	44.6	0.2	43.0
	9	82.3	5.5	1.5	0.7	0.5
Group 3	13	76.3	2.0	32.2	0.3	31.4
	14	78.9	2.0	56.3	0.1	55.7
	15	82.9	6.6	3.2	0.2	0.1

Relative abundance (%): low — high

Supplementary Figure 6. Water content and the proportion of Proteobacteria and Gammaproteobacteria in each fecal sample. The proportion of Proteobacteria and Gammaproteobacteria are shown in highly concentrated fecal samples (0.3 g/ml) in GuSCN solution. DNA was extracted within 3 days and after storage for one month at room temperature.

Group	Sample	*Citrobacter*	*Esherichia*	*Enterobacter*	*Trabulsiella*	*Serratia*	*Klebsiella*	Others/unclasified
Group 1	1	0.0	0.2	0.0	0.0	0.1	0.0	0.2
	2	0.0	0.4	0.0	0.0	0.1	0.0	0.3
	3	0.0	0.3	0.0	0.0	0.2	0.0	0.5
	4	37.3	0.2	8.1	0.8	0.1	0.3	5.5
Group 2	7	0.6	0.0	0.5	0.0	0.0	0.0	1.0
	8	0.0	0.0	20.8	0.4	0.0	4.7	13.5
	9	0.0	0.0	0.0	0.0	0.0	0.0	0.7
Group 3	13	17.8	0.0	8.3	0.5	0.1	0.1	9.0
	14	12.4	0.0	5.0	0.2	0.1	8.9	18.6
	15	0.0	0.0	0.0	0.0	0.0	0.0	0.3

Relative abundance (%): low — high

Supplementary Figure 7. Proportion of dominant genera of Gammaproteobacteria in feces in diluted GuSCN solution.
The heat map shows the genus abundance (%) of Gammaproteobacteria in fecal samples that were stored in GuSCN solution diluted with 1.0 ml of PBS for one month at room temperature.

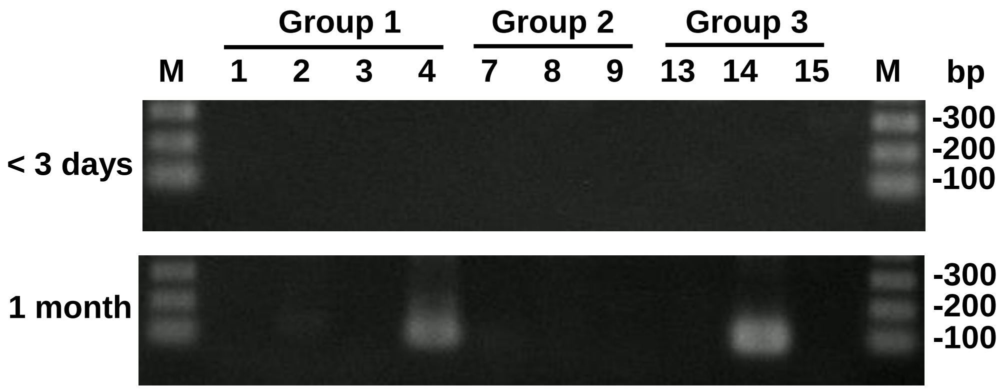

Supplementary Figure 8. Detection of genomic DNA of Citrobacter in highly concentrated fecal samples by PCR. Citrobacter-specific PCR was performed using DNA extracted from fecal samples in diluted GuSCN solution within 3 days and after storage for one month at room temperature. M, 100 bp DNA ladder.

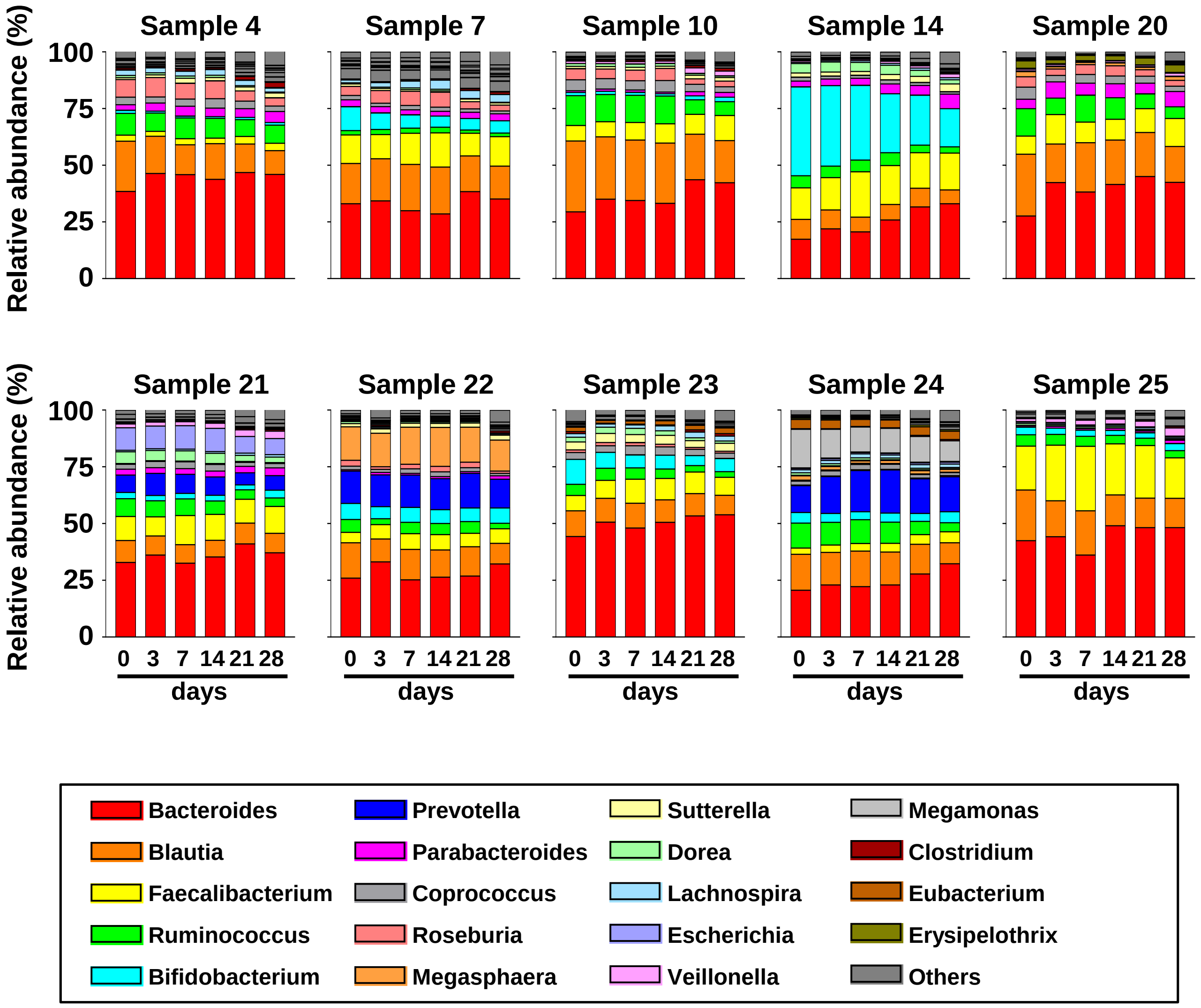

Supplementary Figure 9. Effects of storage term on the microbial composition in feces in GuSCN solution.
Microbial composition at the genus level in fecal samples stored at room temperature. DNA was extracted after storage for 0, 3, 7, 14, 21, and 28 days.

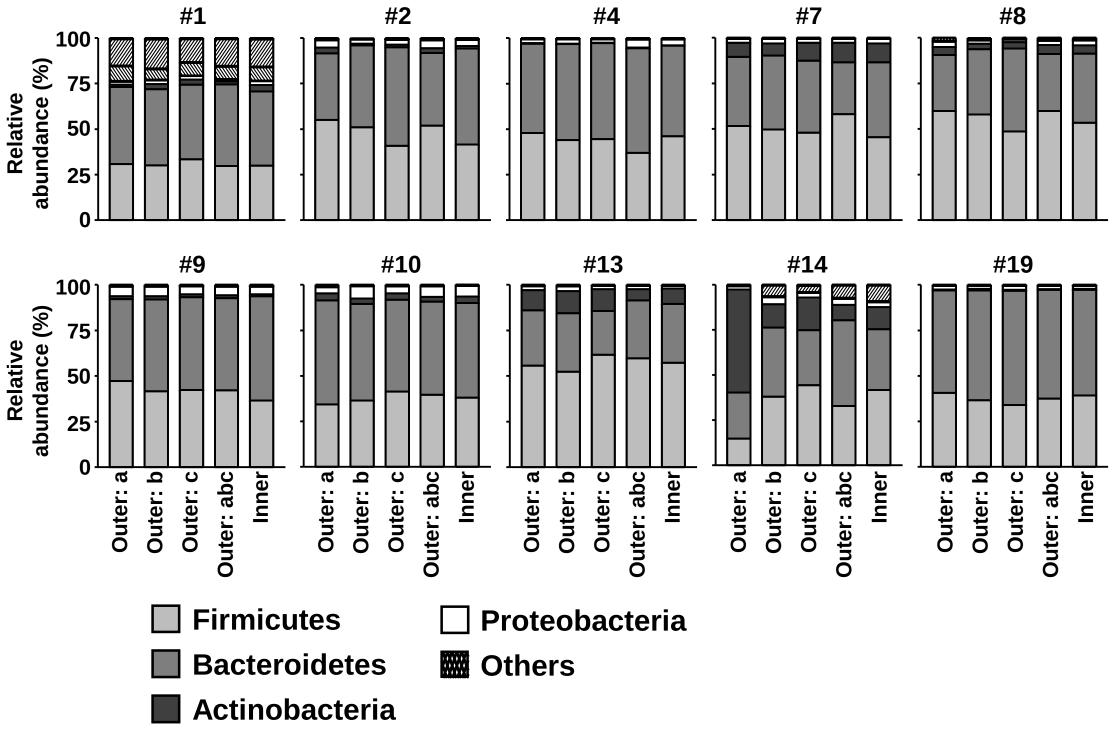

Supplementary Figure 10. Microbial composition at the phylum level from different positions in fecal samples.

Supplementary Table 1. Microbial composition at the phylum level in GuSCN solutions containing different concentrations of feces (0.01–0.3 g/ml)

			< 3 days		1 month				
Group	Sample	Phylum	0.01	0.3	0.01	0.03	0.1	0.3	(g/ml)
1	1	Firmicutes	21.49	14.47	30.57	28.13	25.37	27.12	
		Bacteroidetes	51.67	80.02	40.90	42.15	43.01	41.45	
		Actinobacteria	7.18	1.94	4.95	5.55	4.87	3.98	
		Proteobacteria	1.31	0.16	1.88	2.44	2.39	2.00	
		Others	18.35	3.41	21.70	21.73	24.37	25.45	
	2	Firmicutes	45.85	49.07	50.26	52.04	46.25	42.98	
		Bacteroidetes	44.61	41.13	40.71	42.41	47.72	19.72	
		Actinobacteria	5.81	2.74	3.88	2.51	3.15	3.61	
		Proteobacteria	3.61	6.56	4.94	2.78	2.68	33.37	
		Others	0.12	0.50	0.21	0.25	0.20	0.33	
	3	Firmicutes	39.80	29.14	37.23	42.57	46.53	42.94	
		Bacteroidetes	59.08	68.77	61.40	54.70	50.50	55.70	
		Actinobacteria	0.15	0.14	0.20	0.55	0.60	0.14	
		Proteobacteria	0.37	0.61	0.48	0.90	1.23	0.55	
		Others	0.60	1.34	0.70	1.28	1.14	0.67	
	4	Firmicutes	42.81	32.38	42.22	43.18	47.13	36.12	
		Bacteroidetes	54.72	63.34	54.12	52.47	48.07	3.94	
		Actinobacteria	0.17	0.16	0.19	0.26	0.41	0.22	
		Proteobacteria	2.23	4.02	3.37	3.95	4.19	59.61	
		Others	0.07	0.10	0.10	0.13	0.19	0.11	
2	7	Firmicutes	63.93	63.20	54.84	56.22	43.32	24.71	
		Bacteroidetes	25.30	23.23	36.40	39.27	49.04	17.40	
		Actinobacteria	9.16	12.76	4.33	1.87	2.55	1.40	
		Proteobacteria	1.22	0.67	3.43	2.18	4.06	55.91	
		Others	0.38	0.14	0.99	0.46	1.04	0.57	
	8	Firmicutes	51.15	54.55	53.55	47.93	53.66	34.96	
		Bacteroidetes	40.80	24.99	38.31	44.61	42.67	15.73	
		Actinobacteria	4.30	16.17	3.31	2.65	1.43	3.71	
		Proteobacteria	3.07	2.67	3.90	3.98	1.81	44.56	
		Others	0.68	1.62	0.93	0.83	0.42	1.03	
	9	Firmicutes	45.95	36.45	48.44	50.30	50.09	53.21	
		Bacteroidetes	47.47	56.06	43.99	43.04	43.56	44.53	
		Actinobacteria	2.88	1.54	2.62	2.18	2.09	0.62	
		Proteobacteria	3.42	5.48	4.45	4.04	3.89	1.48	
		Others	0.28	0.46	0.50	0.44	0.37	0.16	
3	13	Firmicutes	44.52	37.45	53.06	50.76	52.83	39.24	
		Bacteroidetes	19.03	41.84	22.26	25.45	33.56	15.41	
		Actinobacteria	34.62	18.58	22.57	20.70	10.31	12.82	
		Proteobacteria	1.70	1.96	2.00	2.75	2.87	32.23	
		Others	0.13	0.16	0.12	0.34	0.43	0.31	
	14	Firmicutes	36.12	37.01	39.81	42.71	40.77	24.40	
		Bacteroidetes	41.31	43.78	37.03	38.91	40.05	13.74	
		Actinobacteria	18.00	16.14	17.70	12.23	14.93	5.04	
		Proteobacteria	3.19	1.96	3.83	4.61	2.94	56.29	
		Others	1.38	1.11	1.63	1.54	1.31	0.53	
	15	Firmicutes	54.29	36.09	54.98	49.28	55.00	52.64	
		Bacteroidetes	36.51	53.76	35.93	43.60	36.78	41.74	
		Actinobacteria	5.38	3.36	4.63	3.48	4.18	2.24	
		Proteobacteria	3.65	6.63	4.07	3.53	3.66	3.21	
		Others	0.17	0.15	0.39	0.11	0.38	0.17	

Supplementary Table 2. Microbial composition at the phylum level for feces in GuSCN solution diluted with PBS

			< 3 days			1 month			
Group	Sample	Phylum	0	0.1	1.0	0	0.1	1.0	PBS (ml)
1	1	Firmicutes	23.11	25.19	27.97	25.37	24.88	31.59	
		Bacteroidetes	53.70	49.05	49.86	43.01	43.69	42.66	
		Actinobacteria	5.96	3.36	5.29	4.87	4.01	4.35	
		Proteobacteria	1.10	0.86	0.94	2.39	1.39	1.20	
		Others	16.13	21.53	15.94	24.37	26.03	20.20	
	2	Firmicutes	28.99	39.82	56.18	46.25	51.23	60.15	
		Bacteroidetes	67.97	56.06	39.31	47.72	41.80	35.16	
		Actinobacteria	1.10	1.60	2.42	3.15	3.13	2.65	
		Proteobacteria	1.80	2.39	1.95	2.68	3.60	1.83	
		Others	0.15	0.13	0.14	0.20	0.23	0.21	
	3	Firmicutes	38.98	36.97	30.95	46.53	46.62	41.28	
		Bacteroidetes	59.80	61.94	67.77	50.50	50.50	55.15	
		Actinobacteria	0.11	0.09	0.13	0.60	0.68	0.82	
		Proteobacteria	0.22	0.22	0.66	1.23	1.07	1.59	
		Others	0.88	0.78	0.48	1.14	1.14	1.17	
	4	Firmicutes	49.78	40.44	41.39	47.13	35.76	15.45	
		Bacteroidetes	46.67	55.14	55.58	48.07	60.64	30.60	
		Actinobacteria	0.21	0.19	0.20	0.41	0.22	0.12	
		Proteobacteria	3.26	4.13	2.74	4.19	3.28	53.72	
		Others	0.08	0.09	0.10	0.19	0.11	0.10	
2	7	Firmicutes	66.03	68.39	68.68	43.32	52.54	43.58	
		Bacteroidetes	29.86	22.72	21.02	49.04	40.59	43.94	
		Actinobacteria	3.04	7.52	8.80	2.55	2.67	5.49	
		Proteobacteria	0.88	1.11	1.18	4.06	3.29	5.27	
		Others	0.18	0.26	0.32	1.04	0.92	1.71	
	8	Firmicutes	51.46	55.99	59.99	53.66	59.61	41.31	
		Bacteroidetes	39.11	24.31	28.75	42.67	33.69	14.92	
		Actinobacteria	6.00	15.21	7.62	1.43	3.70	1.78	
		Proteobacteria	2.74	2.93	2.58	1.81	2.04	41.39	
		Others	0.69	1.56	1.07	0.42	0.96	0.60	
	9	Firmicutes	46.95	44.57	43.80	50.09	46.79	49.75	
		Bacteroidetes	45.50	48.25	49.52	43.56	50.15	42.98	
		Actinobacteria	4.87	2.20	3.21	2.09	1.03	2.57	
		Proteobacteria	2.40	4.66	3.20	3.89	1.88	4.22	
		Others	0.28	0.31	0.28	0.37	0.15	0.47	
3	13	Firmicutes	24.60	30.04	42.61	52.83	54.74	35.85	
		Bacteroidetes	13.71	9.51	16.11	33.56	24.01	12.78	
		Actinobacteria	61.08	59.87	39.80	10.31	18.48	13.32	
		Proteobacteria	0.51	0.50	1.32	2.87	2.39	37.72	
		Others	0.10	0.08	0.16	0.43	0.38	0.32	
	14	Firmicutes	28.29	34.34	39.87	40.77	44.78	37.67	
		Bacteroidetes	13.41	36.04	28.64	40.05	37.95	18.32	
		Actinobacteria	57.67	28.12	29.60	14.93	12.78	9.66	
		Proteobacteria	0.47	1.23	1.46	2.94	3.47	33.66	
		Others	0.16	0.28	0.43	1.31	1.02	0.68	
	15	Firmicutes	43.27	48.54	53.28	55.00	50.87	50.32	
		Bacteroidetes	24.22	29.67	35.60	36.78	40.60	41.25	
		Actinobacteria	31.42	20.35	7.52	4.18	4.06	3.97	
		Proteobacteria	0.98	1.34	3.43	3.66	4.14	4.08	
		Others	0.11	0.10	0.17	0.38	0.32	0.39	
